# Inhibiting Forkhead box K1 induces autophagy to reverse epithelial-mesenchymal transition and metastasis in gastric cancer by regulating Myc-associated zinc finger protein in an acidic microenvironment

**DOI:** 10.18632/aging.103013

**Published:** 2020-04-08

**Authors:** Yixuan Wang, Libin Sun, Wensheng Qiu, Weiwei Qi, Yaoyue Qi, Zhao Liu, Shihai Liu, Jing Lv

**Affiliations:** 1 Department of Oncology, Affiliated Hospital of Qingdao University, Qingdao 266071, Shandong, China; 2 Central Laboratory, Affiliated Hospital of Qingdao University, Qingdao 266071, Shandong, China

**Keywords:** gastric cancer, FOXK1, MAZ, autophagy, EMT

## Abstract

Background: Forkhead box K1 (FOXK1) is a transcription factor belonging to the Forkhead box (FOX) family and is closely related to the development of various cancers, but the functional mechanism through which FOXK1 regulates autophagy and epithelial-mesenchymal transition (EMT) in the acidic microenvironment of gastric cancer (GC) remains unclear.

Results: Our results indicated that the inhibition of FOXK1 induced autophagy and thus exerted antimetastatic effects in an acidic microenvironment. The dual inhibition of mammalian target of rapamycin (mTOR) and FOXK1 enhanced autophagy and reversed EMT of acidic GC cells. In addition, FOXK1 activated transcription in conjunction with the MAZ promoter.

Conclusion: Together, our results suggest that FOXK1 can be used as an independent prognostic indicator for GC patients. We also revealed a new strategy involving the cotargeting of FOXK1 and autophagy to reverse the effects of EMT. MAZ is involved in the development and progression of GC as a downstream target of FOXK1.

Methods: Here, the cellular responses to the inhibition of FOXK1 in GC were studied in vivo and in vitro through wound healing assays, transwell assays, Western blotting, laser confocal microscopy and transmission electron microscopy. The molecular mechanisms of FOXK1 and Myc-associated zinc finger protein (MAZ) were studied via chromatin immunoprecipitation sequencing (ChIP-seq), bioinformatics, Western blotting, and quantitative real-time PCR (q-PCR).

## INTRODUCTION

Gastric cancer (GC) is the fifth most common malignancy in the world and the third leading cause of cancer-related death [1]. According to available statistics, GC kills more than 320,000 people each year in China, which corresponds to 45% of the global death toll [2]. Although advanced GC patients can undergo surgical resection and chemotherapy, the results are unsatisfactory due to problems such as recurrence. Comprehensive treatment for advanced GC is currently not available. Therefore, it is necessary to further clarify the molecular mechanism leading to the invasive malignant behavior of GC. Our research team is dedicated to exploring the metastatic behaviors of GC and focusing on the tumor microenvironment [3]. In recent years, scholars have found that tumor cells utilize glycolysis such that the intracellular pH (pH > 7.2) is higher than the extracellular pH (pH ≈ 6.8) in order to maintain rapid growth and proliferation, even in the presence of oxygen [4]. Other studies have also shown that tumors are present in acidic microenvironments and that GC transfer is a multistep behavior regulated by the acidic microenvironment [5]. Therefore, tumor acidosis is an important factor at all stages of disease development, including growth, invasion, neovascular growth, and genetic instability [6].

Forkhead box K1 (FOXK1) belongs to the Forkhead box (FOX) transcription factor family and plays many important roles in cell cycle regulation, cell proliferation and differentiation, and metabolic regulation [7]. Since the first report of the FOXK1 gene (1994), there has been a certain understanding of the promotion of FOXK1 in tumorigenesis and development. Preliminary studies have investigated the roles of FOXK1 in ovarian cancer, colorectal cancer, and glioblastoma [8–11], but the role of FOXK1 in GC has been less studied. A study conducted by Wu et al. revealed that FOXK1 plays an important role in inducing the invasion and migration of colorectal cells by inducing epithelial-mesenchymal transition (EMT) [12]. EMT is an important event during which a cell undergoes phenotypic changes in embryonic development, tissue remodeling, and wound healing and plays a key role in tumor invasion and metastasis [13]. EMT allows cancer cells to survive independently of the primary tumor site in the absence of a nutritional support system, and these cells are thus prone to undergo autophagy to gain energy [14].

Autophagy is a highly evolutionarily conserved mechanism that captures and degrades aging cytokines and proteins and damaged organelles in vivo to ensure maintenance of the cellular metabolism [15]. Autophagy might be induced under various stresses, including starvation and anoxic and acidic microenvironments. These conditions thus provide cells with energy for the maintenance of cellular homeostasis; thus, autophagy protects cells from an acidic microenvironment [16, 17]. However, the effects of autophagy on cancer cells remain controversial. The role of autophagy in cancer cells appears to depend on the type and stage of the tumor and the intensity of autophagy-induced stimulation [18]. Some studies have shown that autophagy might protect the genome from damage and inhibit tumorigenesis, but this process also activates metabolic stress responses [19, 20]. However, the exact contribution of autophagy to EMT in the acidic microenvironment of GC remains unclear. Studies conducted by Xie et al. have shown that acidic microenvironments can induce autophagy to protect lung cancer cells [21]. Moreover, Gugnoni’s team revealed that autophagy might negatively regulate EMT in papillary thyroid cancer [22]. For this reason, the inhibition of autophagy can reverse EMT. However, the molecular mechanism and exact function of FOXK1 in autophagy under acidic conditions are poorly understood.

Myc-associated zinc finger protein (MAZ), also known as serum amyloid A-activating factor 1 (SAF1), Pur-1 or Zif87, was previously considered a key driver of inflammation in animal models [23]. The dysregulated expression of MAZ was recently associated with malignant tumors, such as breast cancer, thyroid cancer, hepatocellular carcinoma and urothelial carcinoma [24–27]. A growing body of experimental and clinical data indicates that MAZ plays a key role in tumor differentiation, EMT, invasion and metastasis [28, 29]. Isolation of the MAZ protein using a stable K-Ras promoter analog prevents MAZ from activating K-Ras transcription and delays tumor growth in mice. This evidence confirms the prominent role of MAZ in mouse development [30]. In addition, upregulated MAZ regulates prostate cancer proliferation and metastasis through interaction with androgen receptors [31]. However, the underlying mechanisms through which MAZ regulates autophagy and EMT in GC remain unknown.

In this study, we explored the functional significance of inhibiting FOXK1-activated autophagy to regulate EMT in the acidic microenvironment of GC. In addition, we successfully demonstrated that MAZ serves as a downstream factor that directly binds to FOXK1 in GC. Our study indicates that FOXK1 expression is upregulated in primary metastatic GC (mGC) tissues. Moreover, the downregulation of FOXK1 can induce autophagy and contribute to inhibiting metastasis and reversing EMT by downregulating MAZ. Therefore, our study of FOXK1/MAZ in an acidic microenvironment might clarify the processes of autophagy and EMT in GC and thus provides independent prognostic indicators and therapeutic targets for GC.

## RESULTS

### FOXK1 expression is significantly increased in GC metastasis

**Figure 1:**
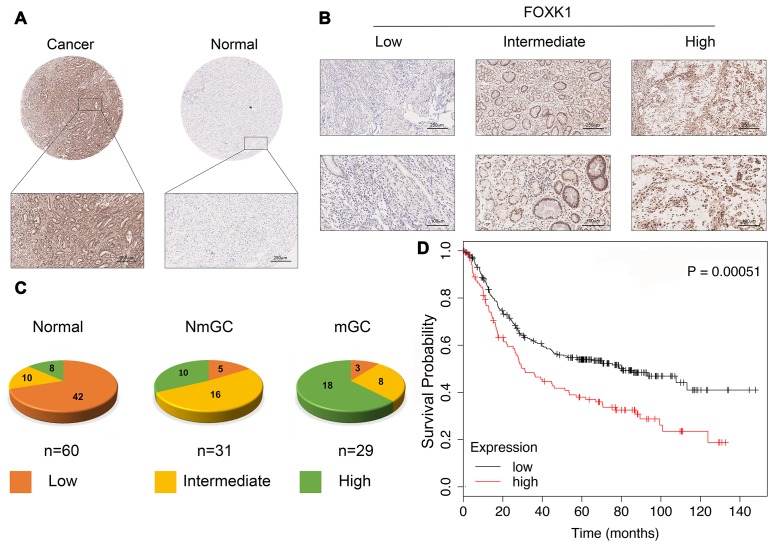
**FOXK1 expression is significantly increased in GC metastasis. (A**) Representative images of FOXK1 TMA analysis in GC tissues and adjacent tissues. Scale bar, 250 μm. (**B**) TMA assay of the levels of FOXK1 protein in GC tissues. Representative images of FOXK1 staining in GC tissues from the TMA are shown. Scale bar, 250 μm and 100 μm. (**C**) Analysis of staining in 60 groups of TMAs. (**D**) Kaplan-Meier analysis of survival rates of GC patients with high FOXK1 expression and GC patients with low FOXK1 expression.

We first verified whether FOXK1 expression is related to GC progression. We analyzed cancerous and adjacent tissues in the Oncomine database and found that FOXK1 was significantly overexpressed in GC in the different datasets (Supplementary Figure 1). To further verify the high expression of FOXK1 in GC, we subjected 60 GC tissues and their adjacent tissues, which included 31 tissues of nonmetastatic gastric cancer (NmGC) and 29 primary metastatic gastric cancer tissues (mGC), to immunohistochemical staining (IHC). Representative images are shown in Figure 1A and 1B. The level of FOXK1 expression in normal tissues adjacent to cancer tissues was significantly lower than that in the cancer tissues. The strongest expression of FOXK1 was found in mGC tissues (Figure 1C). This finding indicates that FOXK1 is highly expressed in GC tissues and is most highly expressed in mGC tissues. A Kaplan-Meier survival analysis revealed that increases in FOXK1 expression were associated with decreased in the patient’s overall survival (OS) (Figure 1D). These results strongly suggest that the upregulation of FOXK1 is closely related to progression and poor prognosis in GC patients.

### FOXK1 downregulation inhibits metastasis in vivo

**Figure 2:**
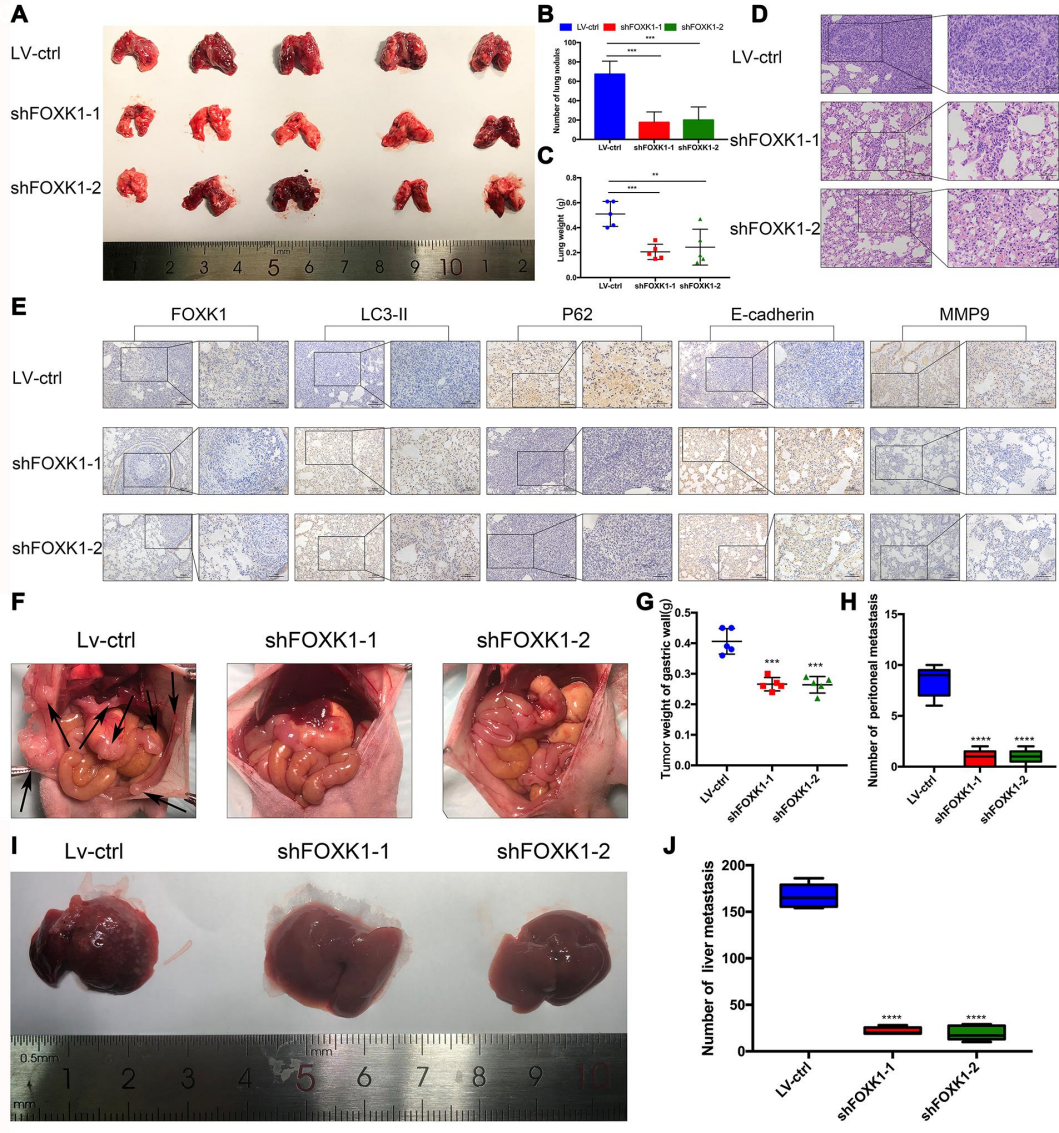
**Downregulation of FOXK1 inhibits metastasis in vivo.** (**A**) Lung tissues were obtained from nude mice injected with differently treated MGC803 cells (n=5). (**B**) The number of lung nodules per nude mouse was determined. (**C**) The lung weight of each nude mouse was calculated. (**D**, **E**) Representative images of H&E staining of lung sections (scale bar, 100 μm and 50 μm) and IHC staining of FOXK1, LC3-II, P62, E-cadherin and MMP9 levels in lung tissue (scale bar, 100 μm and 50 μm). (**F**) MGC803 cells transfected with LV-shFOXK1-1 and LV-shFOXK1-2 were orthotopically transplanted into the stomach walls of nude mice (n=5). (**G**, **H**) Weight of gastric wall tumors (g) and number of metastatic tumors (**H**) at 28 days post transplantation with MGC803 cells. (**I**, **J**) Representative images (**I**) and number (**J**) of diffuse liver metastases. * P < 0.05, ** P < 0.01, *** P < 0.001, and **** P < 0.0001.

To further determine the role of FOXK1 in GC tissues, we assessed whether the silencing of FOXK1 inhibits the lung partialization of diffuse GC cells in vivo and established a mouse tumor model via tail vein injection. As shown in Supplementary Figure 2, we performed Western blotting and qRT-PCR analyses to determine the expression of FOXK1 in GC cells. The expression of FOXK1 was significantly decreased in FOXK1 short hairpin RNA (shRNA)-transfected cells compared with control cells. We transfected MGC803 cells with a lentivirus (LV) encoding shFOXK1-1 and shFOXK1-2. The same number of LV-ctrl, LV-shFOXK1-1 and shFOXK1-2 MGC803 cells were then injected through the tail vein into three groups of 6-week-old BALB/c nude mice. Twenty-eight days after implantation, all the mice were sacrificed, and the metastatic nodules on the surfaces of the lungs were recorded. Significantly reduced lung nodules were found in the LV-shFOXK1-1 and LV-shFOXK1-2 groups compared with the LV-ctrl group (Figure 2A, 2B). In addition, the lung weights of the LV-shFOXK1-1 and LV-shFOXK1-2 groups were less than those of the LV-ctrl group (Figure 2C). Hematoxylin and eosin (H&E) staining also confirmed that the injection of LV-ctrl cells into the tail vein resulted in a significant increase in pulmonary nodules (Figure 2D). In addition, IHC measurements of these metastatic nodules excised from the two groups of mice showed that the silencing of FOXK1 increased the expression of LC3-II and E-cadherin and decreased the expression of P62 and MMP9 compared with the expression levels observed in the control nodules (Figure 2E). These results show that FOXK1 silencing significantly inhibits GC lung metastasis.

Furthermore, we orthotopically transplanted MGC803 cells infected with LV-ctrl, LV-shFOXK1-1 and shFOXK1-2 into the gastric wall of nude mice. Twenty-eight days after orthotopic transplantation, we found that the inhibition of FOXK1 reduced the number of metastases and tumor weight in the gastric wall (Figure 2F–2H). The inhibition of FOXK1 has also been shown to decrease the number of diffuse liver metastases (Figure 2I, 2J). Collectively, the inhibition of FOXK1 in vivo can inhibit GC transfer.

### In an acidic microenvironment, FOXK1 inhibition inhibits the invasion and metastasis of GC cells in vitro

**Figure 3:**
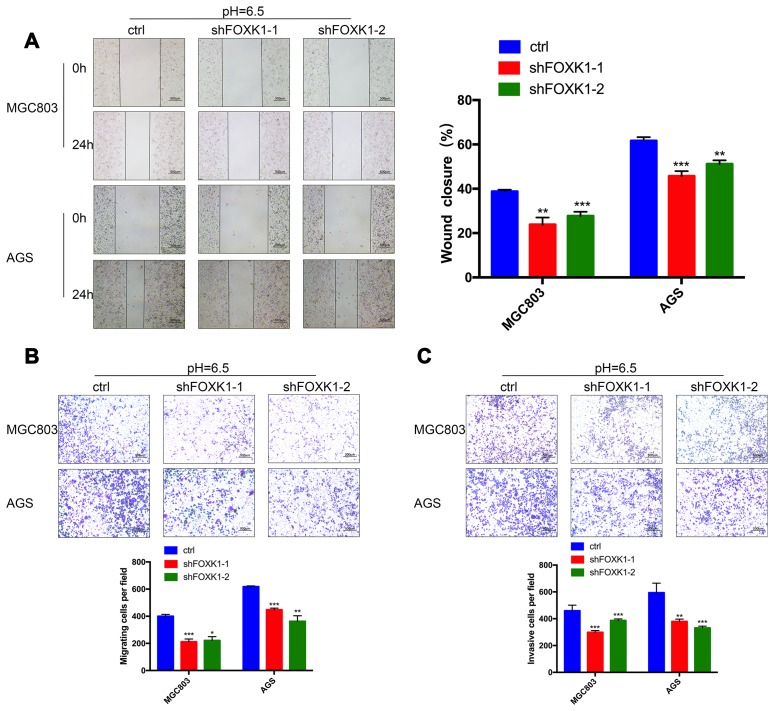
**FOXK1 inhibition inhibits the invasion and metastasis of GC cells in an acidic microenvironment in vitro.** (**A**) Wound healing assays were performed to detect changes in the migration of MGC803 and AGS cells at pH 6.5. The analysis of wound healing was performed using ImageJ software. Scale bar, 500 μm. (**B**) Pretreatment of MGC803 and AGS cells at pH 6.5. Transwell assays were performed to assess the migration capacity of GC cell lines. Scale bar, 500 μm. (**C**) Transwell assays were performed to assess the invasive ability of acidic MGC803 and AGS cells. Scale bar, 500 μm. The data are presented as the means ± S.D.s from three independent experiments. * P < 0.05, **P < 0.01 and *** P < 0.001.

The above-described results indicate that FOXK1 exerts a significant effect on GC metastasis. We subsequently aimed to demonstrate the effect of FOXK1 inhibition on GC cell migration and invasion in vitro. We first verified the function of FOXK1 under normal conditions. As shown in Supplementary Figure 3, compared with the control group, the inhibition of FOXK1 in a normal environment resulted in increased LC3-II expression and decreased P62 expression, which indicated that the inhibition of FOXK1 under normal conditions can induce autophagy. In addition, the inhibition of FOXK1 under normal circumstances can increase the expression level of E-cadherin and suppress the expression of N-cadherin and Vimentin compared with that found in the control group. This finding indicates that the inhibition of FOXK1 under normal conditions inhibits the EMT process. A recent study revealed that the microenvironment of solid tumors is more acidic than that in the surrounding normal tissue [4]. Our team also confirmed that an acidic microenvironment is more prone to promoting the invasion and metastasis of GC cells than the normal environment, which indicates that studying the survival mechanism of GC cells in an acidic microenvironment is of great significance [3]. Interestingly, higher LC3-II, N-cadherin and Vimentin expression but lower P62 and E-cadherin expression was observed under acidic compared with normal conditions (Supplementary Figure 3). These data indicate that autophagy and EMT are more likely to occur in an acidic than in a normal environment. To assess the effect of the extracellular acidic microenvironment on autophagy and EMT in GC cells, we artificially simulated an acidic cellular environment and performed subsequent experiments under these conditions. By analyzing the migration ability of GC cell lines through wound healing assays, we observed that 24 h after the knockdown of FOXK1, the cells exhibited significantly reduced healing ability compared with the control cell lines (Figure 3A). Further transwell experiments confirmed that FOXK1 inhibition effectively reduced the invasion of MGC803 and AGS cells under both Matrigel and Matrigel-free conditions (Figure 3B, 3C). In conclusion, the silencing of FOXK1 in an acidic microenvironment significantly inhibits the migration and invasion of GC cells.

### The inhibition of FOXK1 induces autophagy to inhibit the migration and invasion of acidic GC cells

**Figure 4:**
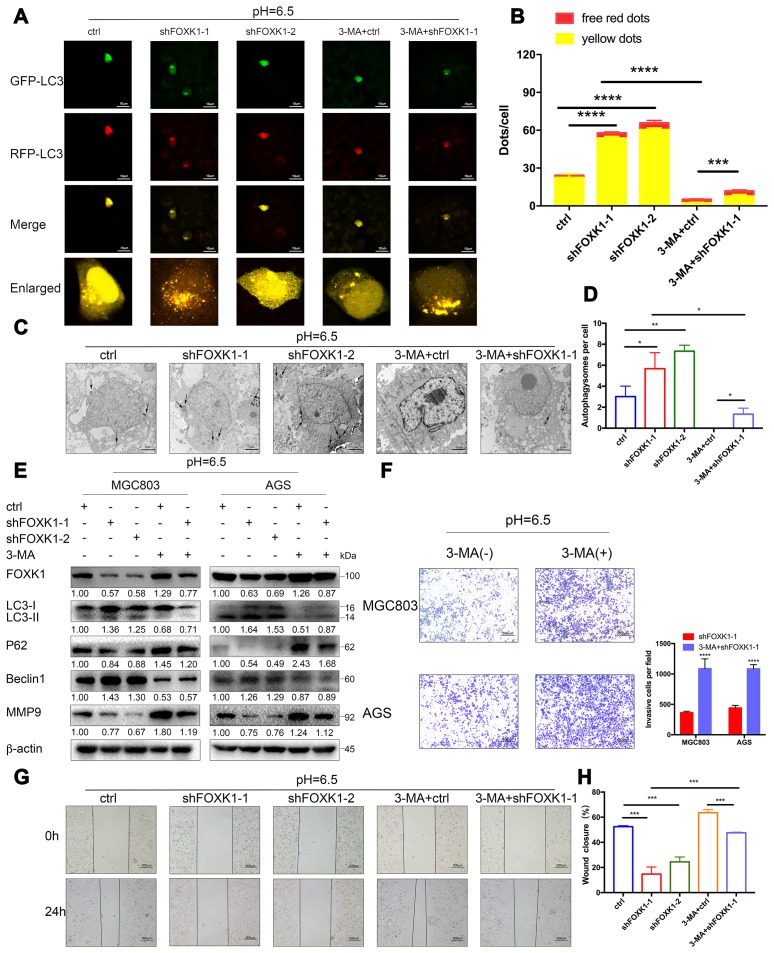
**The silencing of FOXK1 induces autophagy to inhibit the migration and invasion of acidic GC cells.** (**A**–**H**) Acidic MGC803 and AGS cells were infected with an empty lentivirus control (LV-ctrl) or with LV shFOXK1-1 or shFOXK1-2 and subsequently incubated for 24 h prior to pretreatment with 2 mM 3-MA or PBS (control). (**A**, **B**) Laser confocal microscopy analysis and quantification of transfected MGC803 cells with plasmid constructs containing LC3, which was fused with tandem mRFP-GFP tags. Scale bar, 10 μm. (**C**, **D**) The number of autophagosomes was observed and quantified under a transmission electron microscope. Scale bar, 2 μm. (**E**) Western blotting analysis of the levels of FOXK1, LC3-I, LC3-II, P62, Beclin1 and MMP9; β-actin was used as a loading control. (**F**) Matrigel cell invasion assays of MGC803 and AGS cells were performed, and the invading cells were quantified. (**G**, **H**) Scratch test evaluation of MGC803 cells. The wound healing area was analyzed using ImageJ software. Scale bar, 500 μm. The data are presented as the means ± S.D.s from three independent experiments. * P < 0.05, ** P < 0.01, *** P < 0.001, and **** P < 0.0001.

A previous study showed that the FOXK1 and SIN3A complexes inhibit the basal levels of autophagy [32], the association between FOXK1 and autophagy under acidic conditions has not been reported. To elucidate the effect of the inhibition of FOXK1 on autophagic flux, we observed MGC803 cells transiently infected with the adenovirus mRFP-GFP-LC3 under a laser scanning confocal microscope. In this system, the GFP-mRFP-LC3 reporter localized as yellow puncta in autophagosomes and red-only puncta in autolysosomes because the acidic lysosomal environment induces the decay of GFP fluorescence but maintains the red fluorescence of mRFP [33]. As shown in Figure 4A, the MGC803 cells belonging to the control group and transfected with mRFP-GFP-LC3 exhibited the basal levels of autophagy, and the knockout of FOXK1 expression significantly increased the number of red-only LC3 puncta, which indicates an increase in autophagic flux (Figure 4A, 4B). By transmission electron microscopy, we found that autophagosomes infected with shFOXK1-1 and shFOXK1-2 were significantly more autophagic than those belonging to the control group (Figure 4C, 4D). Western blotting further confirmed that the silencing of FOXK1 in MGC803 and AGS cells increased the levels of LC3-II and Beclin1 proteins and decreased the levels of P62 proteins (Figure 4E). The above results indicate that the inhibition of FOXK1 significantly induces autophagy in GC cells at pH 6.5. We subsequently investigated the role of FOXK1-induced autophagy inhibition in GC metastasis. As expected, laser confocal microscopy showed that treatment with the autophagy inhibitor 3-methyladenine (3-MA) significantly reduced the number of red-only LC3 puncta (Figure 4A, 4B). The production of autophagosomes was also reduced, as demonstrated by transmission electron microscopy (Figure 4C, 4D). A Western blotting analysis also showed that 3-MA treatment decreased LC3-II and Beclin1 protein expression, and increased P62 protein expression. (Figure 4E). In addition, cell scratch and transwell experiments confirmed that the inhibition of autophagy by 3-MA antagonized the inhibitory effect of the knockout of FOXK1 on the migration and invasion of GC cells (Figure 4F–4H). Further Western blotting results confirmed that the expression of MMP9 increased after treatment with 3-MA. Together, these results indicate that the inhibition of FOXK1 expression induces autophagy to inhibit migration and invasion.

### MAZ is a new transcription activation target of FOXK1

**Figure 5:**
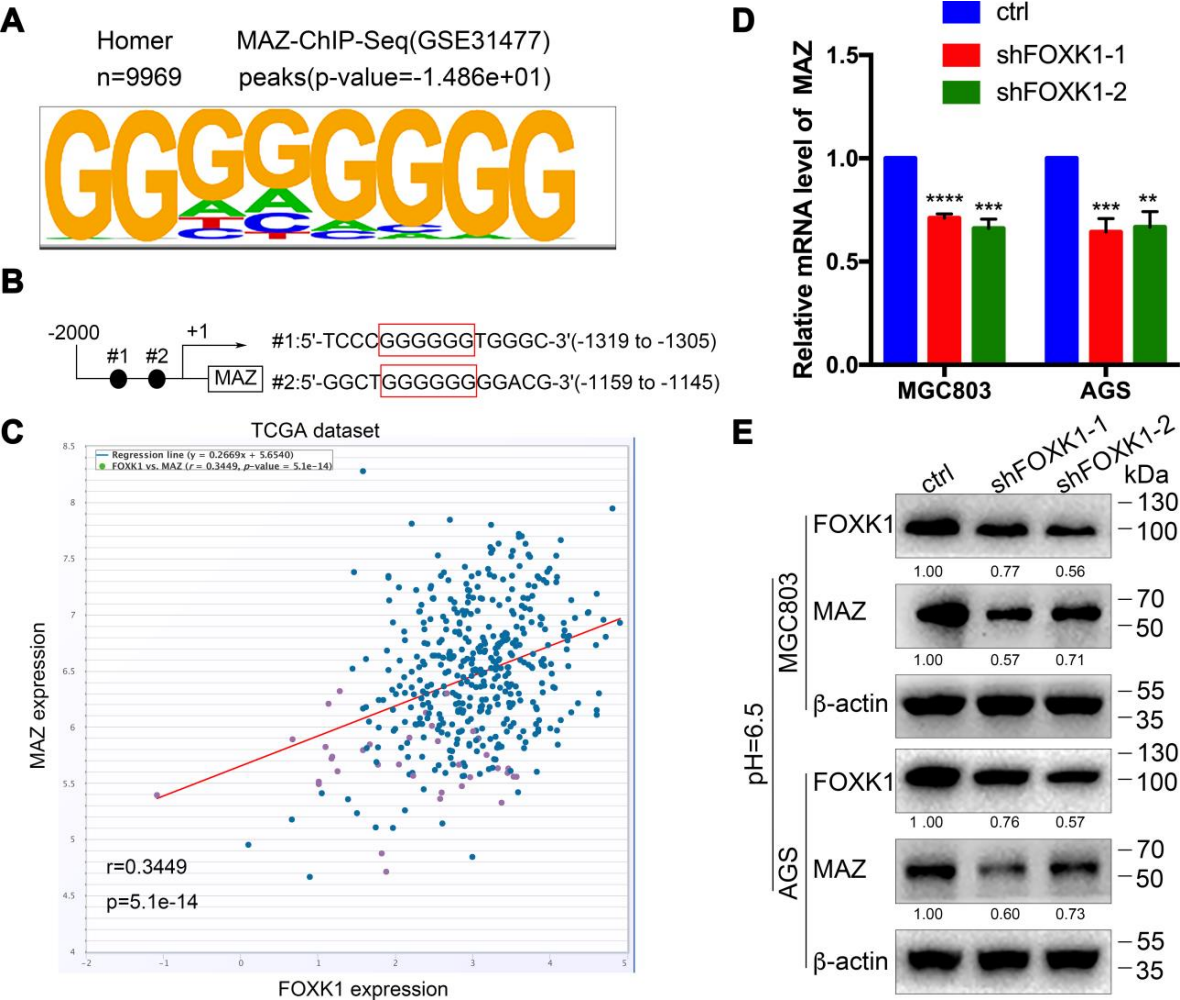
**MAZ is a new transcription activation target of FOXK1.** (**A**) Analysis of the 9969 overlapping FOXK1 loci for overrepresented sequence elements identified significantly enriched FOXK1-like DNA-binding motifs, and the results showed that 12.92% of the FOXK1 genomic loci contained at least one of the motifs. (**B**) Potential binding sites for FOXK1 at the 5'-UTR of MAZ. (**C**) FOXK1 and MAZ coexpression in the TCGA GC dataset was analyzed by querying the open database ChIPBase v2.0 (r: 0.3449, p = 5.1e-14). The mRNA (**D**) and protein (**E**) levels of MAZ in MGC803 and AGS cells were attenuated by the silencing of FOXK1. The data are presented as the means ± S.D.s from three independent experiments. ** P < 0.01, *** P < 0.001, and **** P < 0.0001.

To further decipher the molecular mechanism through which the silencing of FOXK1-induced autophagy promotes GC cell migration and invasion, we sought to identify the direct target of FOXK1 by analyzing the DNA-binding region of endogenous FOXK1 through chromatin immunoprecipitation sequencing (ChIP-seq) using a substantive antibody against FOXK1. As shown in Figure 5A, MAZ exhibited the best match to the consensus GGGGGG motif (E value = -1.486e + 01). Moreover, a bioinformatics analysis demonstrated that two binding sites for MAZ might exist in the 5’-untranslated region (UTR) of FOXK1 (Figure 5B). In addition, the coexpression of FOXK1 and MAZ was analyzed by querying the open database ChIPBase v2.0 in The Cancer Genome Atlas (TCGA) GC dataset. The results showed that FOXK1 was positively correlated with the MAZ gene in GC (r: 0.3449, p = 5.1e-14) (Figure 5C). Based on the bioinformatics results, we performed q-PCR and Western blotting analyses to determine whether FOXK1 exerts an effect on the expression of MAZ in human GC. The forced silencing of FOXK1 led to decreases in MAZ mRNA and protein expression (Figure 5D, 5E). In summary, the above-described experimental results indicate that FOXK1 can activate its transcription by binding to the MAZ promoter.

### Acidic conditions stimulate shFOXK1 to induce autophagy and inhibit EMT through the downregulation of MAZ

**Figure 6:**
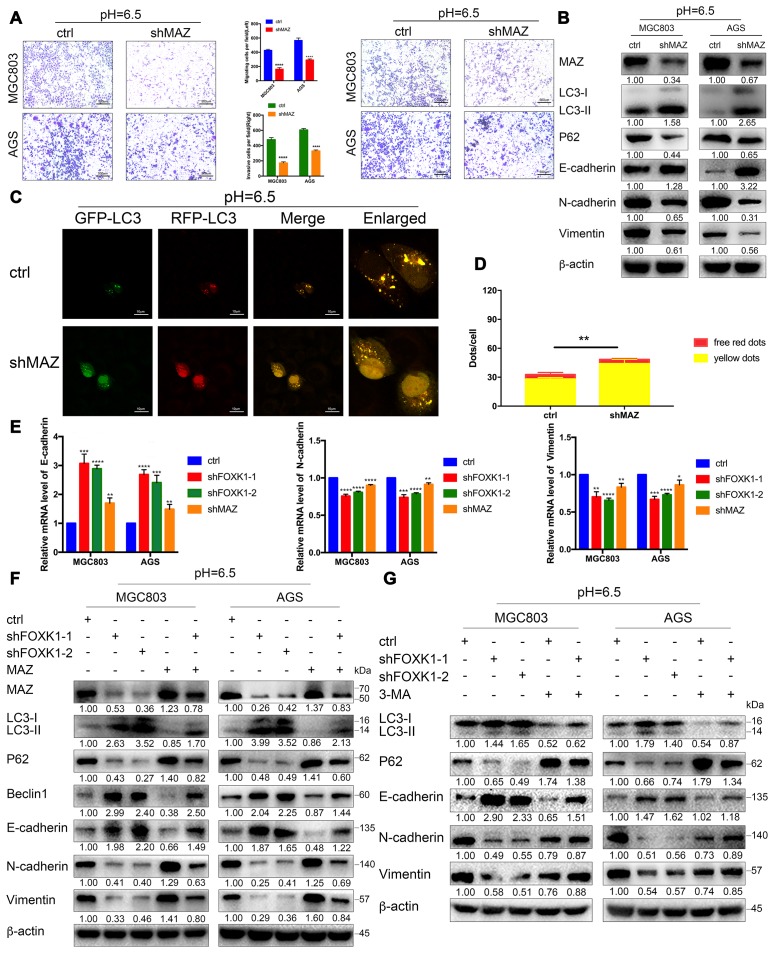
**Acidic conditions stimulate shFOXK1 to induce autophagy and inhibit EMT through the downregulation of MAZ.** (**A**) Transwell assays were performed to assess the migration and invasion of MGC803 and AGS cells following MAZ knockdown at pH 6.5. Scale bar, 500 μm. (**B**) Western blotting was performed to detect the protein expression of MAZ, autophagy-related proteins and EMT-related proteins in GC cells treated with ctrl or shMAZ in an acidic microenvironment. (**C**) Immunofluorescence staining was used to detect the autophagic flux in acidic MGC803 cells treated with ctrl or shMAZ, and the quantified results are shown in (**D**). Scale bar, 10 μm. (**E**) qRT-PCR analysis of EMT-related mRNA expression in MGC803 and AGS cells expressing ctrl, shFOXK1-1, shFOXK1-2 and shMAZ. (**F**) Western blotting was performed to detect the expression of MAZ, autophagy-related proteins and EMT-related proteins in ctrl (or shFOXK1-1)-transfected acidic GC cells cotransfected with MAZ plasmids. (**G**) MGC803 and AGS cells cultured at pH 6.5 were pretreated with 2 mM 3-MA or PBS (control) and then incubated with LV-ctrl, shFOXK1-1 and shFOXK1-2 for 24 h. The expression levels of autophagy-related proteins and EMT-related proteins were verified by Western blotting and quantified. The data are presented as the means ± S.D.s from three independent experiments. * P < 0.05, ** P < 0.01, *** P < 0.001, and **** P < 0.0001.

To clarify the role of MAZ in FOXK1 signaling, we first verified the effects of MAZ on autophagy, EMT, migration and invasion in an acidic microenvironment. Transwell and Matrigel transwell assays showed that the inhibition of MAZ significantly inhibited the migration and invasion of GC cells (Figure 6A). Similarly, a wound healing analysis of cellular migration ability revealed that the inhibition of MAZ significantly reduced the 24-h healing ability of MGC803 and AGS cells compared with that of the control cells (Supplementary Figure 4A). Moreover, our Western blotting results confirmed that E-cadherin expression was significantly increased in shMAZ-transfected acidic MGC803 and AGS cells compared with the control group. In contrast, the expression levels of N-cadherin and Vimentin in shMAZ-transfected acidic MGC803 and AGS cells were significantly reduced compared with those found in the control group (Figure 6B). These data indicate that inhibition of MAZ under acidic conditions can inhibit EMT, migration and invasion of GC cells. Furthermore, immunofluorescence assays showed that the numbers of red-only LC3 puncta were increased in shMAZ-transfected MGC803 cells (Figure 6C, 6D). Western blotting assays showed that the knockdown of MAZ significantly increased the expression level of LC3-II and decreased the expression level of P62 (Figure 6B). The abovementioned results indicate that the inhibition of MAZ significantly induces autophagy in GC cells at pH 6.5.

We subsequently tested whether FOXK1 regulates EMT through MAZ. The knockdown of FOXK1 or MAZ in the MGC803 and AGS cell lines significantly increased E-cadherin mRNA expression but decreased the N-cadherin and Vimentin mRNA levels (Figure 6E). In addition, we performed rescue experiments with MGC803 and AGS cells, and the results from a Western blotting analysis showed that the overexpression of MAZ partially abolished the expression of LC3-II, Beclin1 and E-cadherin in acidic GC cell lines transfected with shFOXK1. In contrast, transfection of the overexpressing MAZ plasmid partially reversed the expression levels of P62, N-cadherin and Vimentin in MGC803 and AGS cells transfected with shFOXK1 (Figure 6F). This result was also confirmed by immunofluorescence analysis. Increasing the expression of MAZ eliminated the sudden increase in red-only LC3 puncta caused by FOXK1 knockdown (Supplementary Figure 4B), which demonstrates that the knockout of FOXK1 induces autophagy and suppresses EMT, at least in part by downregulating MAZ. Because FOXK1 inhibition-induced autophagy contributes to metastasis inhibition, we determined whether the silencing of FOXK1-induced autophagy inhibits EMT by observing the relationship between autophagy marker proteins and EMT marker proteins. As expected, the stable knockdown of FOXK1 increased the E-cadherin protein levels and significantly reduced the expression of N-cadherin and Vimentin (Figure 6G). Interestingly, autophagy was significantly inhibited after addition of the autophagy inhibitor 3-MA, whereas the opposite trends were found for E-cadherin, N-cadherin and Vimentin protein expression. This finding indicates that 3-MA-inhibited autophagy can significantly reverse the inhibition of EMT-related protein changes by inhibiting FOXK1 (Figure 6G). Taken together, these data suggest that the inhibition of FOXK1-induced autophagy inhibits EMT progression in an acidic microenvironment, at least in part by downregulating MAZ.

### Dual inhibition of mTOR and FOXK1 enhances autophagy and exerts cooperative antimetastatic effects on acidic GC cells

**Figure 7:**
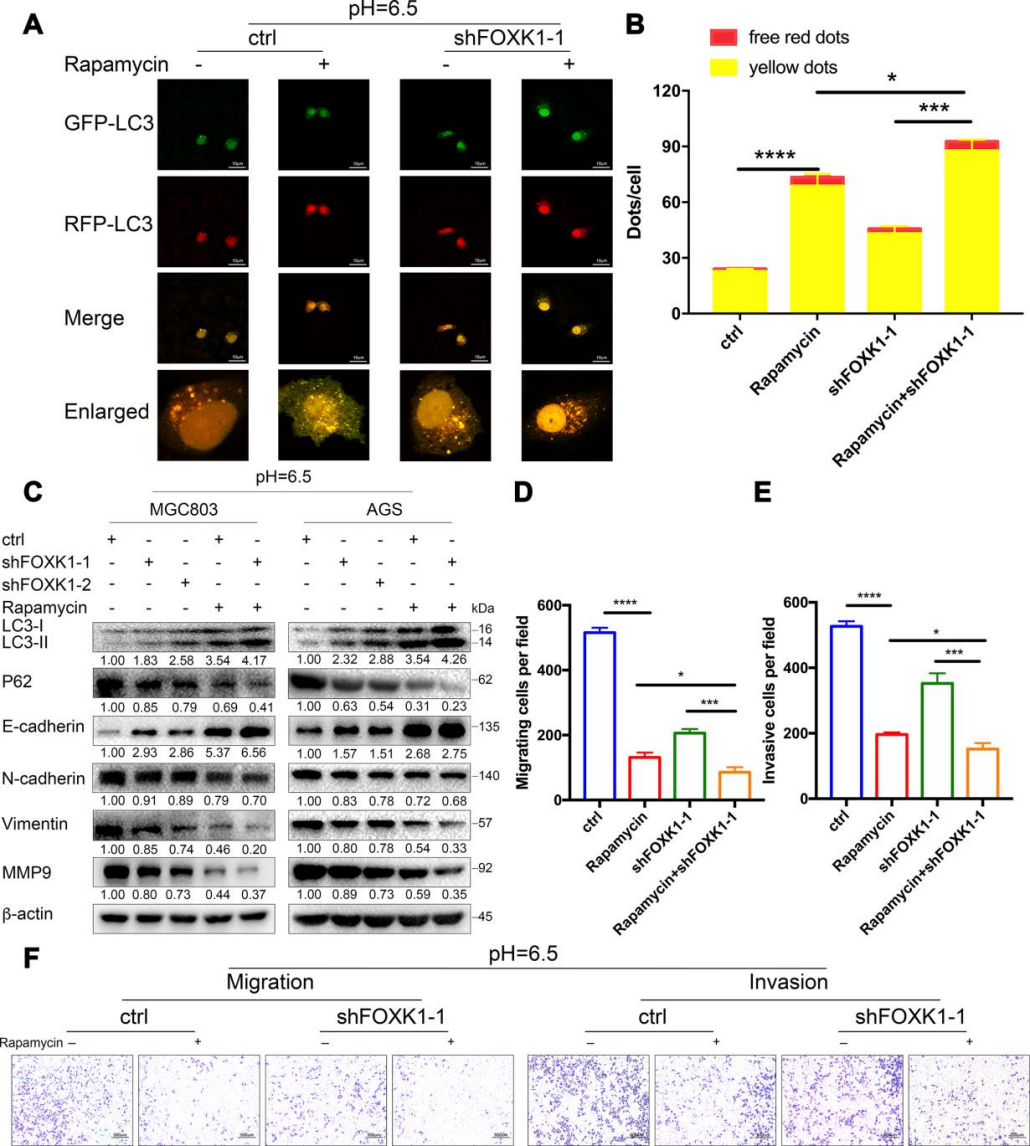
**Dual inhibition of mTOR and FOXK1 enhances autophagy and leads to the synergistic transfer of acidic GC cells.** (**A**–**G**) Acidic MGC803 cells were treated with DMSO or rapamycin (100 nM) or infected with ctrl, shFOXK1-1, shFOXK1-2 or a combination of these for 24 h. (**A**) The red-only and yellow puncta of MGC803 cells transfected with mRFP-GFP-LC3 were observed under laser confocal conditions, and the quantitative results are shown in (**B**). Scale bar, 10 μm. (**C**) Western blotting was performed to assess the expression intensity of LC3-I, LC3-II, P62, E-cadherin, N-cadherin, Vimentin and MMP9. (**D**–**F**) The migration and invasive capabilities of MGC803 cells were examined. The invading cells are quantified in (**D**, **E**). The data are presented as the means ± S.D.s from three independent experiments. * P < 0.05, *** P < 0.001 and **** P < 0.0001.

The above-described results indicate that the inhibition of FOXK1-induced autophagy inhibits the migration and invasion of GC cells in an acidic microenvironment. We further wondered whether the cotargeting of FOXK1 and autophagy could reverse the migration and invasion of GC cells. Because mammalian rapamycin target protein (mTOR) is involved in autophagy regulation and plays an important role in this process, rapamycin is typically used to activate autophagy [34]. First, we examined the effect of the combination of rapamycin and shFOXK1 on the induction of autophagy in MGC803 cells through immunofluorescence. As shown in Figure 7A, treatment with rapamycin or the silencing of FOXK1 resulted in a significant increase in the number of red-only LC3 puncta compared with that of the control group, and their combination exerted a synergistic effect (Figure 7A, 7B). We also performed Western blotting to verify that the conversion of LC3-I to LC3-II was pronounced and that the expression level of P62 was significantly reduced (Figure 7C). We subsequently determined the metastatic ability of MGC803 cells. Interestingly, the superimposed combination of rapamycin and shFOXK1 resulted in a significant reduction in the number of transwell chambers compared with the single treatments in the presence and absence of Matrigel (Figure 7D–7F). We also found that treatment with the combination significantly increased the E-cadherin protein levels compared with the single treatments (Figure 7C). In contrast, treatment with the combination significantly reduced the N-cadherin, Vimentin and MMP9 protein levels compared with the single treatments (Figure 7C). Taken together, these results indicate that interference with the combination of FOXK1 and rapamycin significantly increases the effect on autophagy and suppresses the metastasis of GC cells. Therefore, the above-described studies can provide new clues for targeted autophagy therapy and important ideas for reversing EMT and inhibiting metastasis in GC.

## DISCUSSION

**Figure 8:**
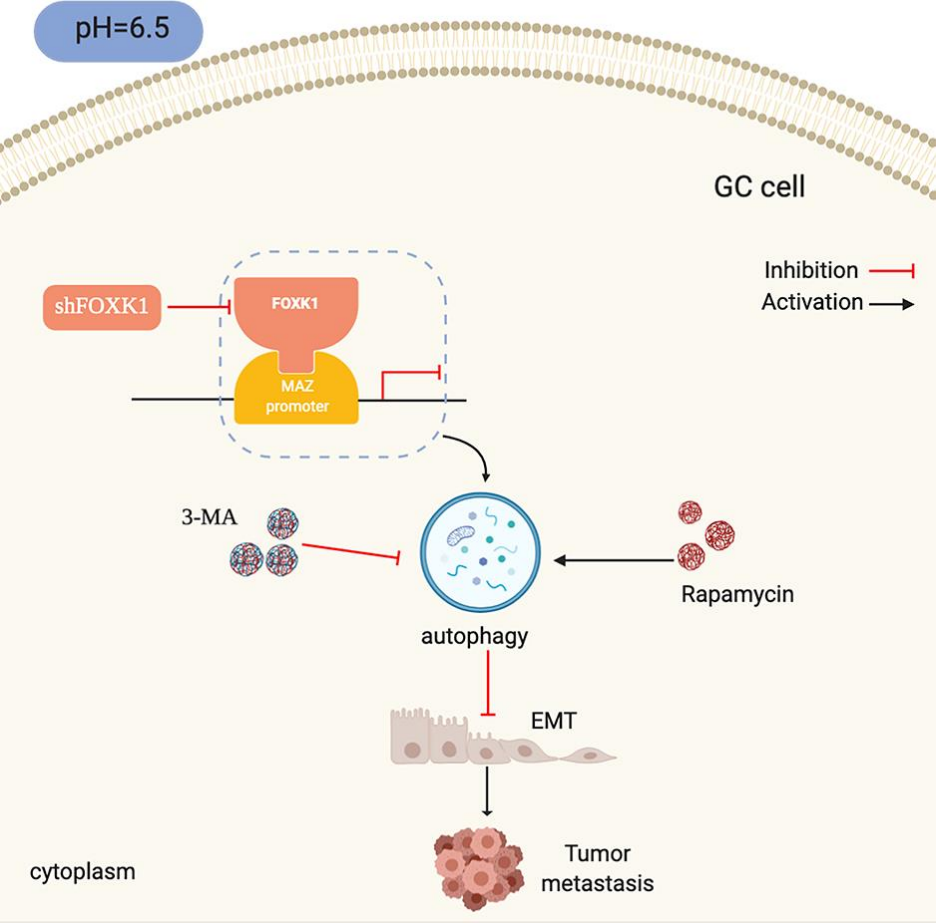
**Schematic diagram depicting the mechanism through which FOXK1-mediated autophagy reverses EMT and thereby inhibits GC metastasis by modulating MAZ in an acidic microenvironment.**

This study investigated the downregulation of the transcription factor FOXK1 in an acidic microenvironment for inhibiting the invasion and metastasis of GC cell lines. We found that FOXK1 is highly expressed in primary mGC tissues, which indicates that FOXK1 is useful for prognosis and serves as a positive predictor of GC recurrence and metastasis. In the present study, we further found that the stimulation of shFOXK1 under acidic conditions could modulate EMT and thereby inhibit the migration and invasion of GC cell lines. Intensive studies have revealed that the silencing of FOXK1-induced autophagy is likely to negatively regulate EMT in an acidic microenvironment. We also explored the role of FOXK1 and MAZ in the development and progression of GC and the underlying mechanisms (Figure 8). To our knowledge, this paper describes the first investigation of the regulation of autophagy by the FOXK1/MAZ axis under GC acidification conditions. Our findings might provide unique insights into the pathogenesis and invasive biology of GC.

This study focused on the key role of FOXK1 in GC transfer in an acidic microenvironment. Tumor cells preferentially undergo glycolysis despite aerobic conditions and thereby produce a large amount of H^+^ [35]. In addition, due to an insufficient blood supply, tumors often have hypoxic areas that promote anaerobic metabolism and lactic acid formation [36]. These conditions can allow GC cells to survive in an acidic microenvironment. The role and function of FOXK1 in cancer have been widely studied, but the metastasis of tumors under acidic conditions is not well understood. Our studies indicate that the downregulation of FOXK1 significantly impairs GC invasion and migration in vitro and in vivo. A previous study revealed that FOXK1 promotes the migration and invasion of SW480 cells [12], and our study supports these results to some extent. We also performed in vivo experiments to verify that FOXK1 can inhibit tumor metastasis.

EMT is a physiological process that increases the ability of cells to invade and metastasize and plays an important in the metastasis and development of many cancers. Due to its role in research, this process can provide a forward-looking perspective for clinical treatment, and the use of the inhibition of EMT as a therapeutic strategy is an exciting topic of research. Previous studies have shown that the coexistence of E-cadherin with N-cadherin and/or Vimentin indicates that the related cells might be in a partial EMT state [37, 38]. Our results indicate that the inhibition of FOXK1 increases E-cadherin expression, reduces N-cadherin and Vimentin expression in an acidic microenvironment and also decrease the expression levels of MMP9. It is believed that the inhibition of FOXK1 can reduce the changes in the invasion and migration abilities of GC cells. Previous studies have also revealed that the coexpression of FOXK1 and Vimentin can upregulate Snail to promote EMT in GC and thereby promotes the metastasis of GC cells in vitro and in vivo [39]. This finding is also consistent with our results, which suggests that the inhibition of EMT might be an important mechanism for inhibiting FOXK1 to attenuate GC metastasis.

Autophagy is a pathway for lysosomal degradation that involves the phagocytizing, digestion, and recycling of proteins and organelles within cells to produce energy [15]. EMT allows cancer cells to survive independently of the primary tumor site and in the absence of a nutritional support system. Therefore, autophagy might mediate EMT progression, and this finding is confirmed by our results. The silencing of FOXK1 promoted the conversion of LC3-I to LC3-II and increased the expression of E-cadherin, which indicated that the inhibition of FOXK1 induces autophagy and inhibits EMT in acidic GC. Interestingly, the inhibition of 3-MA-mediated autophagy significantly antagonized the inhibitory effect of the silencing of FOXK1 on acidic GC cell migration and invasion and extensively inhibited silent FOXK1-mediated EMT. These results again demonstrate that the inhibition of FOXK1-induced autophagy in an acidic microenvironment inhibits EMT in GC. This finding is similar to that found by Lv et al., who reported that the activation of autophagy inhibits EMT by degrading Snail in a breast cancer model [40]. Therefore, autophagy is expected to become a new target for reversing EMT. Elucidating the link between autophagy and EMT is necessary for developing new therapeutic strategies that inhibit metastasis.

Because FOXK1 exerts an effect on autophagy, EMT and migration in GC under acidic conditions, we further explored the molecular mechanisms underlying FOXK1 regulation. The analysis of ChIP-seq data revealed that MAZ, a member of the zinc finger protein family, is involved in the development of GC and is a downstream gene of FOXK1. Further mechanistic studies have shown that MAZ is a direct transcription target of FOXK1. This study provides the first demonstration of the involvement of FOXK1 and MAZ interactions in the progression of metastatic GC. Recent studies have shown that MAZ, as an oncogene, can promote EMT in liver cancer cells [28], but whether the inhibition of MAZ in an acidic microenvironment is involved in the regulation of autophagy and EMT in GC cells is not well known. In this study, we found that the knockdown of MAZ under acidic stimulation can promote autophagy and inhibit EMT. Interestingly, the overexpression of MAZ partially eliminated the induction of autophagy by FOXK1 knockdown in acidic GC cells. Taken together, these data clarify that the FOXK1/MAZ axis can represent an effective therapeutic target for regulating autophagy to reverse gastric cancer EMT progression in an acidic microenvironment. Researchers recently discovered that targeted autophagy can be combined with other treatments to obtain better benefits [41]. Our study found that the combination of mTOR inhibition with FOXK1 inhibition induces autophagy in an acidic environment and that this combination is more effective than the antimetastatic effects observed in cells treated with acidic GC alone. The combination therapy significantly increased E-cadherin expression and decreased the N-cadherin, Vimentin and MMP9 protein levels compared with the results obtained after treatment with either agent alone. This result suggests that the targeting of mTOR and FOXK1 can prevent GC metastasis by reversing EMT.

## CONCLUSIONS

In summary, our data suggest that the inhibition of FOXK1 in an acidic microenvironment activates autophagy to inhibit EMT and thereby the migration and invasion abilities of cells, possibly at least in part by downregulating the transcription of the MAZ gene. Our study also demonstrates the functional significance of FOXK1 upregulation in mGC and proposes a new combination therapy that we anticipate will lay the groundwork for a new strategy for the development of effective options for GC treatment. Of course, our current research still has some limitations. For example, the mechanisms related to the upregulation of FOXK1 and MAZ in GC need to be further explored, and the mechanism underlying the interaction between FOXK1 and MAZ needs further study. In future research, we will focus on the molecular mechanisms related to the FOXK1/MAZ axis.

## MATERIALS AND METHODS

### Tissue microarrays (TMAs)

We collected 60 fresh GC tissues and adjacent tissues for IHC staining and evaluation. The staining intensity was given a score from 0 to 3 based on the following criteria: 0, no staining; 1, weak staining; 2, moderate staining; or 3, strong staining. All individual patients understood the objectives of this study and provided written informed consent. The Medical Ethics Committee of Qingdao University and the Affiliated Hospital of Qingdao University approved the collection of clinical materials for research purposes.

### Cells and culture conditions

MGC803 and AGS cell lines were purchased from the cell bank of the Chinese Academy of Sciences and were cultured in RPMI-1640 medium containing 10% fetal bovine serum (FBS) (Gibco, NY, USA). The cells were placed in an incubator at 37 °C with a CO_2_ concentration of 5%. The pH in the medium was adjusted to 6.5 by the adding NaHCO_3_ and equilibrating the medium overnight in an incubator with 5% CO_2_. The pH of the culture medium was determined prior to each experiment to ensure the stability of the culture microenvironment [42].

### Treatment with acidic media and autophagy-related reagents

The cells were seeded in standard RPMI medium buffered at pH 7.4. The next day, the original medium was replaced with a buffer medium with pH 6.5. After 20 h of culture, the cells were treated with 3-MA and rapamycin for 4 h and then collected for further analysis [42].

### Transfection with lentiviral particles

The cells were seeded at a density of 2 x 10^5^ cells/well in six-well plates, and 2 ml of complete medium was added to each well. The cells were incubated for 24 h and infected with lentiviral particles, and 12 h after infection, the LV-containing medium was replaced with fresh complete medium. The infected cells were then selected with 4 μg/ml puromycin for 96 h. Empty lentiviral vector was used as a control. The lentiviral expression vectors LV-ctrl, LV-shFOXK1-1, LV-shFOXK1-2, LV-shFOXK1-3 and LV-shMAZ were purchased from Shanghai Gene Pharma Company (China).

### Reagents and antibodies

FBS and RPMI-1640 were purchased from Gibco (NY, USA). 3-MA and rapamycin were purchased from MCE (Shanghai, China). Autophagy double-labeled adenovirus (mRFP-GFP-LC3) was purchased from Hanbio Biotechnology Co., Ltd., and antibodies against E-cadherin (#14472), N-cadherin (#13116), Vimentin (#5741), LC3-I/II (#3868), β-actin (#4970) and Beclin1 (#3495S) were purchased from Cell Signaling Technology (Beverly, MA, USA). Antibodies against FOXK1 (ab18196) and MMP-9 (ab38898) were purchased from Abcam (Cambridge, MA, USA), and antibodies against MAZ (NB100-86984) were purchased from Novus (Columbus, MO, USA). The secondary antibodies used in this study included goat anti-mouse IgG-HRP (abs20001) and goat anti-rabbit IgG-HRP (abs20002), both of which are available from Absin (Shanghai, China).

### Transwell migration and invasion assays

Cell invasion was detected using a Matrigel (BD, Franklin Lakes, NJ, USA)-coated BD transwell chamber according to the manufacturer’s instructions, and cell migration was detected using a BD transwell chamber without Matrigel. After transfection, 2x10^5^ cells in serum-free medium were added to the upper chamber of the transwell, and medium containing 20% FBS was added to the lower chamber. After culture for 24 h, the cells were fixed with 4% paraformaldehyde for 30 min, stained with crystal violet for 20 min and washed with phosphate-buffered saline (PBS). The cells in five fields under a microscope (up, down, center, left, and right) were counted.

### Wound healing assays

The GC cell lines were seeded into six-well plates and scratched using a sterile pipette tip. After washing with PBS, the cells were incubated in medium containing 2% FBS. Images were obtained at 0 and 24 h under a phase contrast microscope. The percentage of wound healing was measured as follows: [1 - (empty area X h/empty area 0 h)] × 100%.

### RNA isolation and quantitative real-time RT-PCR

Total RNA from cells was isolated using TRIzol reagent (TaKaRa, Beijing, China) and reverse transcribed into cDNA using the PrimeScript RT Master Mix (Perfect Real-Time) reagent (TaKaRa, Beijing, China) according to the manufacturer’s instructions. Quantitative real-time PCR (qRT-PCR) was performed with an ABI 7500HT Fast Real-Time PCR System (Applied Biosystems, CA, USA) and followed by a melting curve analysis. The average relative fold change in mRNA expression was determined using the 2^-ΔΔCt^ method with GAPDH as an internal control. The primer sequences for qRT-PCR were as follows: FOXK1 forward, 5'-GCCACAAAGGCTGGCAGAATT-3', and reverse, 5'-TGGCTTCAGAGGCAGGGTCTAT-3'; MAZ forward, 5'-GCCCTACAACTGCTCCCACT-3', and reverse, 5'-CCGTGGTGAAGCCTTTGTTG-3'; E-cadherin forward, 5′-AAAGCTAGCATGGGCCCTTGGAGCCGCAGCCTC-3′, and reverse, 5′-CGTTTAAACCTAGTCGTCCTCGCCGCCTCCGTA-3′; N-cadherin forward, 5′-CATCCTGCTTATCCTTGTGCTG-3′, and reverse, 5′-CTGGTCTTCTTCTCCTCCACCTT-3′; Vimentin forward, 5′-AAGGAGGAAATGGCTCGTCAC-3′, and reverse, 5′-CTCAGGTTCAGGGAGGAAAAGT-3′; and GAPDH forward, 5'-GGAAGCTTGTCATCAATGGAAATC-3', and reverse, 5'-TGATGACCCTTTTGGCTCCC-3'.

### Protein separation and Western blot assays

GC cells were seeded into 6-cm dishes, treated for 48 h and collected with cell scraping. The cells were fully lysed using RIPA cell lysis reagent containing protease and phosphatase inhibitors (Solarbio, Beijing, China) for 30 min. The cell lysate was aspirated at 12,000 g and 4 °C for 20 min, and the protein concentration was determined using a BCA protein assay kit (Beyotime, Shanghai, China). The supernatant containing the total protein was then mixed with the corresponding volume of 5× SDS loading buffer, and the mixture was heated at 95 °C for 5 min. Subsequently, 20 μg of total protein from each sample was concentrated in a 12% preformed gel. A constant current of 300 mA was used for the wet transfer of the proteins to a 0.22-μm polyvinylidene fluoride (PVDF) membrane. The membrane was blocked with 5% skim milk powder in TBST for 2 h and incubated overnight with the appropriate primary antibody (1:1000). The next day, the membrane was subjected to three 10-min washes with TBST. The membrane was incubated with an HRP-conjugated secondary antibody (1:8000) for 2 h at room temperature and subjected to three 10-min washes with TBST. The bound antibodies were visualized using a chemiluminescence kit (Life Technologies, Shanghai, China) under a Bio-Rad gel imager infrared imaging system (ChemiDoc XRS+).

### Immunofluorescence

The different treated cells were inoculated onto slides for 24 h. The slides were fixed with 4% paraformaldehyde for 20 min, permeated with 0.3% Triton X-100 for 15 min, incubated with 5% bovine serum albumin (BSA) for 30 min and stained with DAPI for 10 min. The slides were then air dried and stored in sealed tablets. The expression of red and green fluorescence was observed under a laser confocal microscope (Leica, Brussels, Belgium). The numbers of red-only LC3 puncta and yellow in three independent replicates were calculated.

### Transmission electron microscopy

The treated cells were centrifuged using a cell scraper, collected and fixed overnight with 2.5% phosphate-buffered glutaraldehyde. The cells were then embedded, sectioned, double-stained with uranyl acetate and lead citrate, and analyzed by transmission electron microscopy.

### ChIP-seq

ChIP-seq was performed using the standard ChIP-seq protocol to detect the localization of DNA-binding proteins in MGC803 cells [43], with slight modifications. Briefly, 10-20 million MGC803 cells were cross-linked with 1% formaldehyde for 10 min in the medium and quenched with 125 mM glycine for 10 min at room temperature. The cells were washed three times with PBS and collected, and ChIP lysis buffer, PMSF, and protease inhibitor were added to the cross-linked MGC803 cells. The cells were then were lysed on ice for 20 min. The tube was placed on ice for ultrasound (25% power, 5-s impact (ice bath), 10-s gap (ice bath cooling), 5~15 min). The chromatin fragment was in the range of approximately 0.1 to 1 kb. The supernatant was centrifuged at 12,000 rpm for 10 min. Immunoprecipitation was then performed overnight at 4 °C with antibodies against FOXK1, and immunoprecipitation, elution and washing were subsequently carried out. DNA product purification was performed using a DNA purification recovery kit, and the resulting product was sequenced using an Illumina HiSeq PE100.

### Immunohistochemistry (IHC) and evaluation of immunostaining

Lung tissues of BALB/c nude mice were collected, fixed in formalin and embedded in paraffin to obtain formalin-fixed, paraffin-embedded (FFPE) tissue samples, and 4-μm-thick sections were then prepared for IHC. All slides were dewaxed and dehydrated, and the endogenous peroxidase activity was then quenched with 3% hydrogen peroxide for 10 min. Antigen retrieval was achieved by covering the slides with citrate buffer (pH 6.0) and heating for 10 min at 95 °C. The sections were then incubated with 10% goat serum albumin for 2 h at room temperature to block nonspecific binding and then incubated with FOXK1, LC3-II, P62, E-cadherin and MMP9 antibodies. After overnight incubation at 4 °C and three washes with PBS, the sections were incubated with the secondary antibody for 1 h at room temperature and rinsed in PBS. Diaminobenzidine (DAB) was used as a chromogen, and the sections were counterstained with hematoxylin. Specimen incubated with PBS instead of the primary antibody served as a negative control. Brown particles present in the cytoplasm and/or nucleus were considered positive signals. The intensity was graded according to a three-point scale: 1, low expression; 2, medium expression; and 3, high expression. The scores were given by two pathologists.

### In vivo experimental metastasis models

Fifteen 6-week-old male BALB/c nude mice were purchased from Beijing Vital River Laboratory Animal Technology Co., Ltd. (Beijing, China). Stable MGC803 cells (LV-ctrl, LV-shFOXK1-1 and LV-shFOXK1-2) were concentrated to 1x10^6^/100 μl in PBS and injected into the nude mice via the lateral tail vein. After 28 days, all lungs from the nude mice were excised and photographed. The numbers and weights of the metastatic tumors in each lung were calculated and recorded. Finally, lung tissue was collected, embedded, fixed and prepared for H&E and IHC staining.

### Orthotropic implantation

An orthotropic mouse model was used to verify the effect of FOXK1 on GC metastasis in mice [44]. Six-week-old male BALB/c nude mice were purchased from Beijing Vital River Laboratory Animal Technology Co., Ltd. (Beijing, China). MGC803 cells (1×10^6^/100 μl) mixed with Matrigel were transplanted into the gastric wall of each mouse (n=5). Twenty-eight days after transplantation, the mice were sacrificed and dissected, and the distributions of the tumor peritoneum and liver metastasis were examined. The tumor weights and numbers of metastases were determined and recorded.

### Statistical analysis

SPSS 19.0 (SPSS, Inc., IL, USA) was used for all statistical tests. The data are presented as the means ± S.D.s and were compared by Student's t test or analysis of variance. P < 0.05 was considered significant.

## Supplementary Material

Supplementary References

Supplementary Figures
