# Supplementary material for: Inhibiting Forkhead box K1 induces autophagy to reverse epithelial-mesenchymal transition and metastasis in gastric cancer by regulating Myc-associated zinc finger protein in an acidic microenvironment
Source: Aging (Albany NY). 2020 Apr 8;12(7):6129–50. doi: 10.18632/aging.103013 (PMC7185099; doi:10.18632/aging.103013)
Supplement: Supplementary References [file aging-12-103013-s001..pdf]

SUPPLEMENTARY REFERENCES

1. Chen X, Leung SY, Yuen ST, Chu KM, Ji J, Li R, Chan AS, Law S, Troyanskaya OG, Wong J, So S, Botstein D, Brown PO. Variation in gene expression patterns in human gastric cancers. Mol Biol Cell. 2003; 14:3208–15. https://doi.org/10.1091/mbc.e02-12-0833 PMID:12925757

2. Cho JY, Lim JY, Cheong JH, Park YY, Yoon SL, Kim SM, Kim SB, Kim H, Hong SW, Park YN, Noh SH, Park ES, Chu IS, et al. Gene expression signature-based prognostic risk score in gastric cancer. Clin Cancer Res. 2011; 17:1850–7. https://doi.org/10.1158/1078-0432.CCR-10-2180 PMID:21447720

3. Hisano M, Erkek S, Dessus-Babus S, Ramos L, Stadler MB, Peters AH. Genome-wide chromatin analysis in mature mouse and human spermatozoa. Nat Protoc. 2013; 8:2449–70. https://doi.org/10.1038/nprot.2013.145 PMID:24232248
