# Supplementary material for: Inhibiting Forkhead box K1 induces autophagy to reverse epithelial-mesenchymal transition and metastasis in gastric cancer by regulating Myc-associated zinc finger protein in an acidic microenvironment
Source: Aging (Albany NY). 2020 Apr 8;12(7):6129–50. doi: 10.18632/aging.103013 (PMC7185099; doi:10.18632/aging.103013)
Supplement: Supplementary Figures [file aging-12-103013-s002..pdf]

SUPPLEMENTARY FIGURES

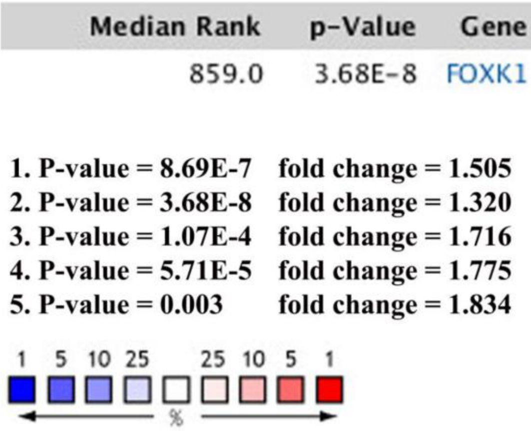

Supplementary Figure 1. Oncomine analysis of gastric cancer vs normal tissue of FOXK1. FOXK1 gene expression in gastric carcinoma samples vs normal tissues. 1. Diffuse Gastric Adenocarcinoma vs.Normal, Chen, Gastric, Mol Biol Cell, 2003 [1]; 2. Gastric Intestinal Type Adenocarcinoma vs Normal, Chen, Gastric, Mol Biol Cell, 2003[1]; 3. Diffuse Gastric Adenocarcinoma vs Normal, Cho Gastric, Clin Cancer Res, 2011 [2]; 4. Gastric Intestinal Type Adenocarcinoma vs Normal, Cho Gastric, Clin Cancer Res, 2011 [2]; 5. Diffuse Gastric Adenocarcinoma vs Normal DErrico Gastric, Eur J Cancer, 2009 [3].

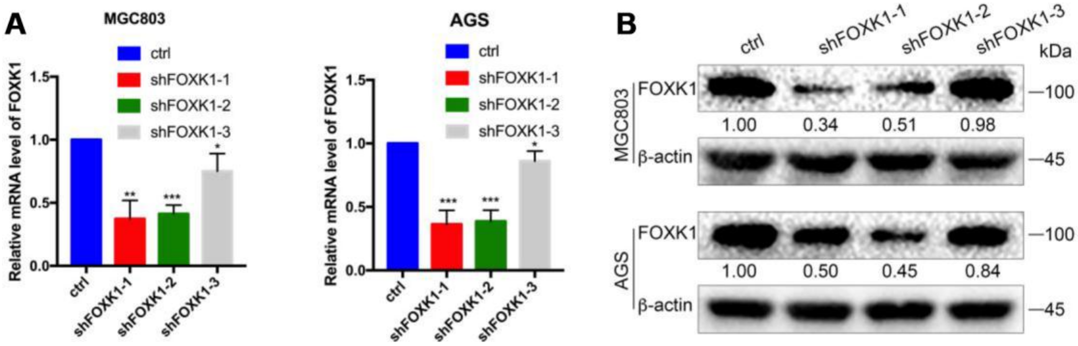

Supplementary Figure 2. Expression of FOXK1 in GC cell lines. (**A**). FOXK1 mRNA expression levels in GC cells infected with LV-ctrl, shFOXK1-1, shFOXK1-2 and shFOXK1-3. (**B**) Infection efficiency of FOXK1-knockdown constructs, as measured by Western blotting. The data are presented as the means ± S.D.s from three independent experiments. * $P < 0.05$, ** $P < 0.01$ and *** $P < 0.001$.

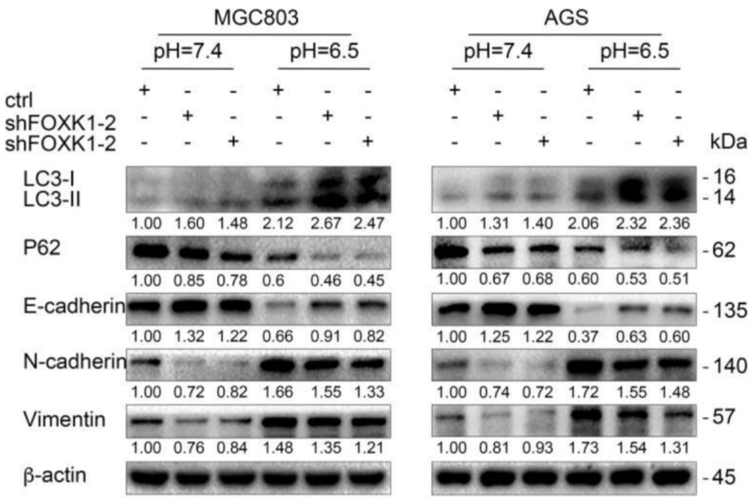

Supplementary Figure 3. Western blotting was performed to analyze the expression levels of autophagy-related proteins and EMT-related proteins in MGC803 and AGS cells with low FOXK1 expression in a low-pH microenvironment. The data are presented as the means ± S.D.s from three independent experiments.

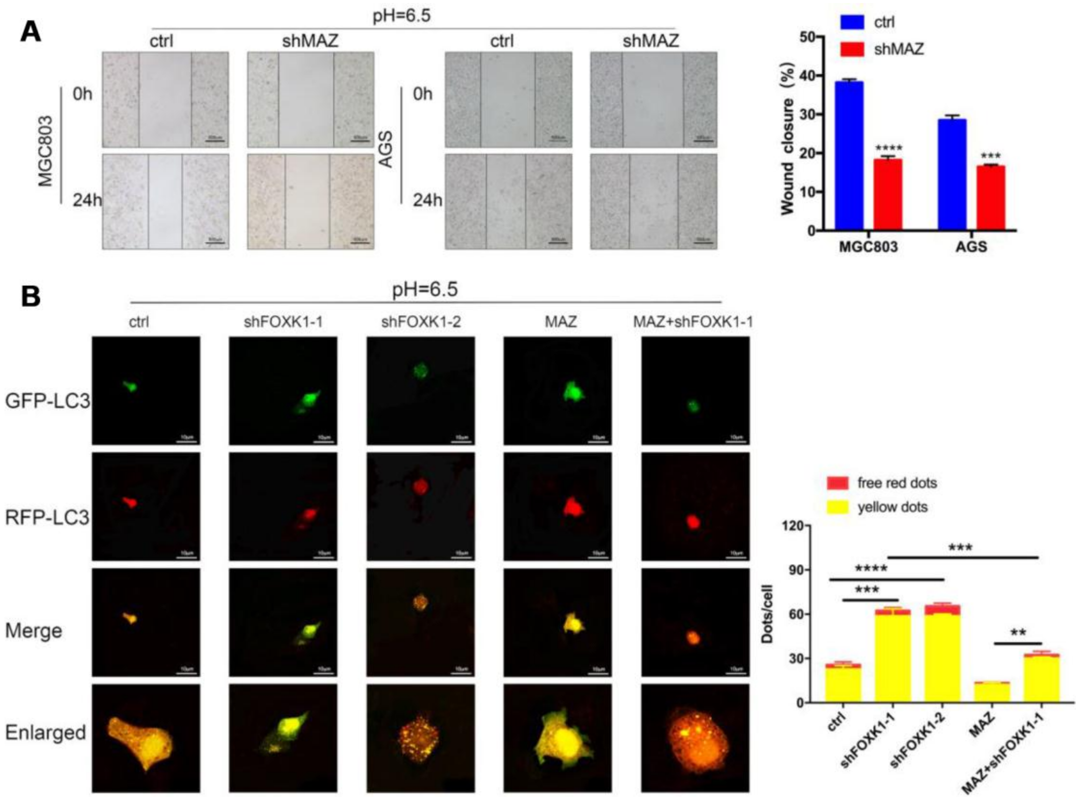

Supplementary Figure 4. Acidic conditions stimulate shFOXK1 to induce autophagy and inhibit EMT through the downregulation of MAZ. (**A**) Scratch test evaluation of MGC803 and AGS cells transfected with ctrl and shMAZ. The wound healing area was analyzed using ImageJ software. Scale bar, 500 μm. (**B**) Immunofluorescence staining was performed to detect the autophagic flux in ctrl (or shFOXK1-1)-transfected acidic MGC803 cells cotransfected with MAZ plasmids. Scale bar, 10 μm. The data are presented as the means ± S.D.s from three independent experiments. ** P < 0.01, *** P < 0.001, and **** P < 0.0001.
